# Relating Health Locus of Control to Health Care Use, Adherence, and Transition Readiness Among Youths With Chronic Conditions, North Carolina, 2015

**DOI:** 10.5888/pcd13.160046

**Published:** 2016-07-21

**Authors:** Meaghan Nazareth, Jordan Richards, Karina Javalkar, Cara Haberman, Yi Zhong, Eniko Rak, Nina Jain, Maria Ferris, Miranda A.L. van Tilburg

**Affiliations:** Author Affiliations: Meaghan Nazareth, Jordan Richards, Karina Javalkar, Yi Zhong, Eniko Rak, Nina Jain, Maria Ferris, The University of North Carolina, Chapel Hill, Chapel Hill, North Carolina; Cara Haberman, Wake Forest University, Winston-Salem, North Carolina.

## Abstract

**Introduction:**

Health locus of control refers to the belief that health is in one’s control (internal control) or is not in one’s control (external control). Among adults, external locus of control is associated with negative health outcomes, whereas internal locus of control is associated with favorable outcomes. Few studies examined these associations among youths. The objective of our study was to determine how locus of control relates to health care use, medication adherence, missed school, and readiness for transition to adult medical care for youths with chronic conditions.

**Methods:**

Participants at a camp for youths aged 6 to 17 years with chronic health conditions completed a survey measuring locus of control, readiness for transition to adult care, and medication adherence. Their parents completed a separate part of the survey about health care use and missed school days in the past year.

**Results:**

A total of 163 youths completed the survey (78.5% white; 52.1% female; mean age, 12.3 y). Internal locus of control (β = 0.196; *P* = .013) and external Doctor locus of control with doctors controlling disease (β = 0.181; *P* = .025) were positively associated with transition readiness. External control by chance or with others controlling disease was negatively associated with transition readiness (β = −0.248; *P* = .002) and positively associated with emergency department visits (β = 0.225; *P* = .004) and with number of hospital inpatient nights at hospital (β = 0.166; *P* = .04).

**Conclusion:**

Adolescents with external control of their health by chance or by other people are at increased risk for negative health outcomes and may fail to develop the self-management skills needed for successful transitioning to adult care. Future studies should examine effects of changes in locus of control on health outcomes among youths.

## Introduction

Health locus of control (LOC) reflects people’s beliefs about who or what is responsible for management of their health condition (1). LOC may influence a person’s health behaviors and can therefore influence health outcomes. In adults, internal LOC (the belief that a person can control his or her health condition and that health-related outcomes are contingent on a person’s behaviors and actions) has been associated with positive health outcomes such as reduced use of emergency departments (EDs) (2) and reduced disease burden and increased self-rated health (3), adherence to treatment (4), and general health (5). Internal LOC may similarly affect youths with chronic conditions, but studies are lacking. Conversely, external LOC indicates that a person believes that outside factors such as doctors, other people, or chance determine health outcomes. In the adult population, external LOC has been associated with negative outcomes, such as decreased quality of life (6), increased ED visits (2), and decreased acceptance of illness (7). A 1985 study found that many children had a strong external LOC and felt that their doctor rather than they themselves were in control of their health condition. This belief was linked to a reduction in independent health decision-making (8), but no data are available suggesting how external LOC affects disease outcomes among youths. Youths with high external LOC may be at a particular risk for negative health outcomes because of dependence on others (eg, doctors, parents, another responsible adult) to manage their condition or because of the belief that chance controls progression of their disease. These 2 factors may hinder learning important self-management skills to prepare for transition to adult health care (9).

Understanding how LOC is associated with outcomes in this population may provide a basis for interventions aimed at changing young patients’ LOC. Health LOC has been a target for interventions in several adult populations, such as women with postpartum depression (10) and people with chronic pain (11). Given the evidence that LOC can be changed and the potential of such interventions to improve health outcomes, the baseline association of LOC and health outcomes among youths should be examined.

Despite the numerous health outcomes associated with LOC among adults, few outcomes have been studied in youths with chronic conditions. The aim of our study was to examine the relationship between LOC and transition readiness, self-management, medication adherence, health care use, and missed school days among youths with chronic conditions. We hypothesized that, as with adults, internal LOC would be associated with improved health outcomes and transition readiness and external LOC would be associated with poor health outcomes and transition readiness.

## Methods

Our study population consisted of youths aged 6 to 17 years who attended Victory Junction Camp in Randleman, North Carolina, during the summer of 2015. Victory Junction is a therapeutic camp that serves youths in this age group with at least one chronic condition such as cancer, sickle cell disease, spina bifida, chronic kidney disease, or diabetes. Most participants came from the southeastern United States. Before the start of camp, we sent an invitation to participate in our study and a link to the online consent forms and survey via email to the parents of each camper. All questionnaires were administered by using the web-based Qualtrics survey software (Qualtrics LLC). Parents gave informed consent and youths gave assent within the Qualtrics platform before they completed the surveys. To be included in the study, both the camper and a parent had to complete their respective portions of the survey. We excluded families in which only a parent or only the child provided data. Parents were asked to report their child’s number of missed school days, ED visits, hospitalizations, and number of inpatient nights spent in the hospital during the past year. Parents provided camper age, sex, race, and insurance status. The study was approved by the institutional review board of the University of North Carolina at Chapel Hill. 

The Multidimensional Health Locus of Control Scale (MHLC) Form C is an 18-item validated questionnaire that assesses a patient’s LOC (12). One subscale of the MHLC is for internal LOC (patients have control over their own health) and 3 are for external LOC (Doctors external, Chance external, and Others external [eg, parents, other responsible adult control health]). The MHLC consists of statements that the camper is asked to agree or disagree with on a 5-point Likert scale, with 1 being “strongly disagree” and 5 being “strongly agree.” The total score for each subscale was calculated by summing the points associated with responses within each subscale. Statements include, for example, “If my condition worsens, it is my own behavior which determines how soon I will feel better again” and “As to my condition, what will be will be.”

We used the University of North Carolina STAR_x_ scale (Self-Management and Transition from Pediatric to Adult-Focused Health Care with R_x_/Treatment scale) as a measure of transition readiness (13,14). This 18-item validated questionnaire was used to collect self-reported transition readiness scores from patients. Six subdomains were defined: communication with physician, engagement during appointments, medication management, disease knowledge, health responsibilities, and resource use. All items were measured on a 5-point Likert scale with the overall scale score as a sum of the point value associated with each response, for a total score ranging from 0 to 90. Examples of questions included were “How often did you make an effort to understand what your doctor told you?” and “How often did you take your medicines on your own?”

The 4-item Morisky Medication Adherence (MMA) scale (15) was used to determine medication adherence. Questions were answered in a yes or no format. The number of “no” answers was summed to create a total score of 1 to 4 with 0 indicating low adherence and higher scores indicating increased adherence. Examples of questions were “Do you forget to take your medicine?” and “Are you careless at times about taking your medicine?”

All analyses were performed using IBM SPSS Version 23 (IBM Corp). Descriptive statistics were used to determine average scale scores and sample characteristics. The Shapiro-Wilk test was used to determine normality of the variables, and variables that were non-normal were transformed by using base-10 logs. Linear regressions were used with domains of the MHLC as independent variables, and age, sex, race, and insurance type were used as control variables. Separate analyses were run for the following 6 dependent variables: transition readiness (using STAR_x_), adherence (using MMA), ED visits, hospitalizations, length of hospital stay, and missed school days. Additionally, we examined the relationship between number of daily medications or weekly injections and LOC.

## Results

We enrolled 163 parent–camper pairs in this study. Surveys were sent to all families with email access, for a total of 903 parent–camper pairs; 260 parents and 176 campers completed surveys. Only those campers whose parents also completed their surveys were included in the sample. The sample was predominantly white (78.5%), and 65% of youths were privately insured. Boys (47.9%) and girls (52.1%) were approximately equally represented, and youths reported various chronic medical conditions with diabetes (19%) being the most common (Table 1).

**Table 1:** Characteristics of Study Population (N = 163) of Youths Aged 6 to 17 Years With Chronic Conditions, North Carolina, 2015

Characteristic	N (%)
**Race/ethnicity**	
White	128 (78.5)
African American	25 (15.3)
Other	10 (6.1)
**Sex**	
Female	85 (52.1)
Male	78 (47.9)
**Insurance**	
Private	106 (65)
Public	28 (17)
Private and Public	28 (17)
No insurance	1 (0.6)
**Age, y, mean (SD)**	12.32 (2.6)
**Diagnoses (more than 2 patients)**	
Diabetes	31 (19.0)
Cerebral palsy	14 (8.6)
Kidney disease	11 (6.7)
Spina bifida	11 (6.7)
Sickle cell anemia	8 (4.9)
Hemophilia	6 (3.7)
Inflammatory bowel disease	6 (3.7)
Down syndrome	3 (1.8)
Cleft palate	3 (1.8)

The mean number of daily medications was 2.48 (standard deviation [SD], 2.72). The mean number of weekly injections was 0.93 (SD, 2.03). An average of one ED visit (SD, 2.0), less than 1 hospital admission (SD, 1.2), 4.3 inpatient nights in the hospital (SD, 9.9), and 11 school absences (SD, 17.6) were reported by parents for the past year. The average transition scale score was 61.9 (SD, 12.9). These variables were nonnormally distributed, and all had positive skewness greater than 2. The data were transformed by using base-10 logs, and after transformation, skewness was within the normal limit (<2). Medication adherence (mean, 2.33; SD, 0.57) was normally distributed and not transformed.

Linear regressions were used to test our hypotheses that LOC is associated with health care use, medication adherence, and transition scores (Table 2). The models predicting medication adherence (F_8,162_ = 1.307, *P* = 0.25, R^2 ^= .07), and missed school (F_8,162 _= 1.490, *P* = .17, R^2 ^= .07), were not significant. The model with transition readiness scores as the dependent variables was significant (F_8,162_ = 2.944, *P* = .004; R^2 ^= 0.133). In that model, internal LOC (β = 0.196, *P* = .01) and Doctors external LOC (β = 0.181, *P* = .03) and others external LOC (β = −0.248, *P* = .002) were associated with transition scores. ED visits (F_8,162_ = 2.305, *P* = .02, R^2 ^= 0.107) were positively associated with Chance external LOC (β = 0.225, *P* = .004). The number of inpatient nights spent in the hospital (F_8,162_ = 3.162, *P* = .002, R^2 ^= 0.141) were associated with an Others external LOC (β = 0.166, *P* = .04). Hospital admissions were not significantly associated with LOC.

**Table 2:** Linear Regressions of Locus of Care (LOC) on Health Care Use, Missed School, Medication Adherence, and Transition Readiness Among Youths Aged 6 to 17 Years With Chronic Conditions (N = 163), North Carolina, 2015

Dependent Variable	Independent Variable	Unstandardized Regression Coefficient (B)^a^	Standard Error	Standardized Regression Coefficient (β)^b^	*P *Value
Emergency department visits^c^	Internal	−0.001	0.003	−0.032	.69
	External, chance	0.009	0.003	0.225	.004
	External, doctors	0.010	0.007	0.117	.15
	External, others	0.000	0.006	−0.006	.94
Hospitalizations	Internal	−0.004	0.002	−0.149	.06
	External, chance	−0.002	0.002	−0.058	.46
	External, doctors	0.001	0.005	0.014	.86
	External, others	0.010	0.005	0.160	.05
Inpatient nights in hospital	Internal	−0.008	0.006	−0.106	.18
	External, chance	0.006	0.006	0.084	.27
	External, doctors	0.002	0.013	0.010	.90
	External, others	0.025	0.012	0.166	.04
Missed school	Internal	0.001	0.007	0.010	.91
	External, chance	0.013	0.007	0.146	.07
	External, doctors	0.013	0.016	0.070	.40
	External, others	−0.007	0.015	−0.038	.64
Transition readiness	Internal	0.003	0.001	0.196	.01
	External, chance	−0.002	0.003	−0.128	.10
	External, doctors	0.006	0.003	0.181	.03
	External, others	−0.008	0.003	−0.248	.002
Medication adherence	Internal	−0.129	0.102	−0.109	.21
	External, chance	−0.033	0.022	−0.130	.13
	External, doctors	−0.054	0.118	−0.039	.65
	External, others	−0.073	0.075	−0.083	.34

a A 1-unit increase in LOC predicts a change of B units in the outcome (dependent variable). Unstandardized regression coefficients in the same model cannot be directly compared.

b For a 1-standard deviation change in LOC domain, the standard deviation of the outcome changes by β units. Standardized regression coefficients in the same model can be compared with higher values indicative of larger associations between LOC domain and outcome.

c Race, sex, age, and insurance coverage were controlled for in each model.

Daily medications were regressed onto each LOC domain and were not significant in any of the domains. When weekly injections were regressed with the LOC domains, significance was found between injections and internal LOC (β = 0.641, *P* = .005) and between injections and Doctors external LOC (β = 0.214, *P* = .046).

## Discussion

This study examined the relationship between LOC and various health outcomes among youths with chronic diseases. A stronger internal LOC or Doctors external LOC corresponded with improved transition readiness, whereas an external others LOC or external chance LOC was associated with decreased transition readiness. This finding suggests that youths who feel they can control the outcome of their disease and do not let their disease course depend on people other than their physician are more likely to learn self-management skills needed for transitioning to adult care. The positive association between Doctors external LOC and increased transition readiness probably indicates that people trust their physician to act in their best interest and thus adhere more strongly to disease management tasks recommended by their physician. Additionally, an increased Chance external LOC was associated with increased ED visits in the past year, whereas an increased Others external LOC was associated with an increase in the number of inpatient nights spent in the hospital. This suggests that youths who believe that other people or chance have a greater effect on their disease progression than they themselves do are more likely to use additional health services than youths with a lower external LOC.

Why we did not observe a significant effect of LOC on school absences or medication adherence is not clear. Other factors may provide a more powerful explanation. For example, parents rather than children will determine if the child stays home from school. Additionally, a large number of youths (28.9%) in our sample did not use medications for their condition. When we limited our analysis to only those participants who did use medication, LOC still was not associated with adherence (F_8,112_ = 1.145, *P* = .34, R^2^ = 0.081). Parents play a key role in their child’s medication adherence (16,17), which may explain why the child’s LOC did not influence adherence. More studies are needed to test the association between LOC and adherence among youths.

Although daily medications were not associated with LOC, weekly injections were. This may be due to the wide variation of conditions and disease severity present in our sample. Our findings demonstrated a positive association between the number of weekly injections and an internal and Doctors external LOC, that is, the more injections a patient performed per week, the greater their internal and Doctors external LOC. In our analyses conducted for this study, we found that an increase in both of these LOC domains were related to improved transition readiness. Because a greater proportion of the youths who completed surveys had diabetes, this relationship between LOC and injections may suggest these youths have stronger internal and Doctors external LOCs, and thus may be more prepared to transition to adult care. This finding may also suggest that patients who regularly have injections may have more opportunities to take their care into their own hands by learning self-injection and are then able to observe the positive outcomes that come with regular injections. Conversely, patients with a higher internal or Doctors external LOC may be more likely to perform their recommended daily injections. This relationship should be explored in future studies.

Health LOC was not previously studied in youths with chronic diseases or in relation to the range of health outcomes that we explored. As among the adult population, a stronger internal LOC was linked to improved outcomes (2–5), whereas stronger external LOCs were related to poor outcomes, such as more hospitalizations. As with adults, an increased external LOC among youths was linked to increased use of EDs (2). Thus, LOC may have similar effects in youths and adults. The effect of parents as an LOC on outcomes among youths needs to be studied.

This study had several limitations. First, all measurements were collected at the same time and no causality can be determined from the study. We could not determine if LOC influences outcomes or if outcomes influence LOC. Second, health care use and school absences were collected by retrospective self-report for the past year. Issues with recall may bias these measurements. Therefore, future studies should include more objective measurements extracted from medical and school records. Third, the sample, although fairly sizeable and varied in medical diagnoses, was largely homogeneous in race and socioeconomic class. Most participants were white and privately insured. Studies with more diversity in race and socioeconomic status are needed. Finally, although Victory Junction campers come from throughout the country, most campers were from the southeastern United States. A broader sample could explore whether geographic location within the United States is associated with LOC or with the health outcomes we studied.

This is the first study of youths with chronic disorders showing the importance of LOC in predicting health outcomes and transition readiness. A stronger internal LOC was associated with improved outcomes, and a stronger external LOC was linked to poorer outcomes. Health care providers can use LOC to identify patients who are at risk for negative outcomes so that steps can be taken to improve outcomes. Evidence suggests that LOC can be a target for intervention. For example, in a sample of adult patients with chronic pain, internal health LOC was increased after the implementation of a pain management program (11), indicating that with increased opportunities to learn about disease management, patients may feel more empowered and in control of their condition. Youths’ health beliefs are not yet fully formed; therefore, they may be more receptive to an intervention that aims to modify LOC. An increase in youths’ internal LOC may correspond with improved health behaviors. Ultimately, this shift in beliefs may significantly improve health outcomes, and adaptation of an intervention targeting LOC for youths may be an important next step.

## References

[1] Wallston KA , Wallston BS . Who is responsible for your health? The construct of health locus of control. In: Sanders G, Suls J, editors. Social psychology of health and illness. Hillsdale (NJ): Lawrence Erlbaum and Associates; 1982. p. 65–95.

[2] Theofilou P . Quality of life and mental health in hemodialysis and peritoneal dialysis patients: the role of health beliefs. Int Urol Nephrol 2012;44(1):245–53. 10.1007/s11255-011-9975-0 21547466

[3] Mautner D , Peterson B , Cunningham A , Ku B , Scott K , LaNoue M . How Multidimensional Health Locus of Control predicts utilization of emergency and inpatient hospital services. J Health Psychol 2015. Epub 2015 Oct 1. 26430065 10.1177/1359105315603468

[4] Basinska MA , Andruszkiewicz A . Health locus of control in patients with graves-basedow disease and hashimoto disease and their acceptance of illness. Int J Endocrinol Metab 2012;10(3):537–42. 10.5812/ijem.3932 23843816 PMC3693625

[5] Berglund E , Lytsy P , Westerling R . The influence of locus of control on self-rated health in context of chronic disease: a structural equation modeling approach in a cross sectional study. BMC Public Health 2014;14(1):492. 10.1186/1471-2458-14-492 24885619 PMC4070405

[6] Taher M , Safavi Bayat Z , Niromand Zandi K , Ghasemi E , Abredari H , Karimy M , Correlation between compliance regimens with health locus of control in patients with hypertension. Med J Islam Repub Iran 2015;29:194–190. 26157712 PMC4476223

[7] Gale CR , Batty GD , Deary IJ . Locus of control at age 10 years and health outcomes and behaviors at age 30 years: the 1970 British Cohort Study. Psychosom Med 2008;70(4):397–403. 10.1097/PSY.0b013e31816a719e 18480188

[8] Perrin EC , Shapiro E . Health locus of control beliefs of healthy children, children with a chronic physical illness, and their mothers. J Pediatr 1985;107(4):627–33. 10.1016/S0022-3476(85)80038-X 4045613

[9] Ferris ME , Mahan JD . Pediatric chronic kidney disease and the process of health care transition. Semin Nephrol 2009;29(4):435–44. 10.1016/j.semnephrol.2009.03.018 19615564

[10] Moshki M , Baloochi Beydokhti T , Cheravi K . The effect of educational intervention on prevention of postpartum depression: an application of health locus of control. J Clin Nurs 2014;23(15-16):2256–63. 10.1111/jocn.12505 24329943

[11] Coughlin AM , Badura AS , Fleischer TD , Guck TP . Multidisciplinary treatment of chronic pain patients: its efficacy in changing patient locus of control. Arch Phys Med Rehabil 2000;81(6):739–40. 10.1016/S0003-9993(00)90103-5 10857516

[12] Wallston KA , Stein MJ , Smith CA . Form C of the MHLC scales: a condition-specific measure of locus of control. J Pers Assess 1994;63(3):534–53. 10.1207/s15327752jpa6303_10 7844739

[13] Ferris M , Cohen S , Haberman C , Javalkar K , Massengill S , Mahan JD , Self-management and transition readiness assessment: development, reliability, and factor structure of the STAR_x_ questionnaire. J Pediatr Nurs 2015;30(5):691–9. 10.1016/j.pedn.2015.05.009 26209873

[14] Cohen SE , Hooper SR , Javalkar K , Haberman C , Fenton N , Lai H , Self-management and transition readiness assessment: concurrent, predictive and discriminant validation of the STAR_x_ questionnaire. J Pediatr Nurs 2015;30(5):668–76. 10.1016/j.pedn.2015.05.006 26165785

[15] Morisky DE , Green LW , Levine DM . Concurrent and predictive validity of a self-reported measure of medication adherence. Med Care 1986;24(1):67–74. 10.1097/00005650-198601000-00007 3945130

[16] Anderson BJ , Vangsness L , Connell A , Butler D , Goebel-Fabbri A , Laffel LM . Family conflict, adherence, and glycaemic control in youth with short duration Type 1 diabetes. Diabet Med 2002;19(8):635–42. 10.1046/j.1464-5491.2002.00752.x 12147143

[17] Ellis DA , Podolski CL , Frey M , Naar-King S , Wang B , Moltz K . The role of parental monitoring in adolescent health outcomes: impact on regimen adherence in youth with type 1 diabetes. J Pediatr Psychol 2007;32(8):907–17. 10.1093/jpepsy/jsm009 17426045

